# Blood inflammatory markers combined with tumor markers for differentiating benign prostatic hyperplasia from prostate cancer

**DOI:** 10.3389/fmed.2026.1730818

**Published:** 2026-02-04

**Authors:** Zeqiang Liu, Xinji Yang, Xiaofei Hou, Peng Du, Keke Jia

**Affiliations:** 1 Department of Laboratory Medicine, Peking University Third Hospital, Beijing, China; 2 Key Laboratory of Carcinogenesis and Translational Research, Ministry of Education, Beijing, China; 3 Department of Urology, Peking University Third Hospital, Beijing, China; 4 Department of Urology, Peking University Cancer Hospital and Institute, Beijing, China

**Keywords:** Aggregate Index of Systemic Inflammation, benign prostatic hyperplasia, diagnostic model, prostate cancer, Systemic Immune-Inflammation Index

## Abstract

**Background:**

Prostate cancer (PCa) is the second most frequently diagnosed malignancy in men worldwide, whereas benign prostatic hyperplasia (BPH) is among the most prevalent non-malignant prostatic disorders. Differentiating between these two conditions remains challenging because of overlapping clinical manifestations and the limited specificity of currently available biomarkers.

**Methods:**

This retrospective study enrolled 200 male patients (53 PCa, 147 BPH) who underwent prostate biopsy or surgical resection based on pathological diagnosis. Clinical characteristics, laboratory parameters, and inflammatory indices were compared between the groups. Both univariate and multivariate logistic regression analyses were conducted to identify independent predictors. Nomograms were developed and validated through calibration curves, ROC analysis, and decision curve analysis (DCA).

**Results:**

PCa patients had significantly higher levels of inflammatory markers than BPH patients (*p* < 0.05). In multivariate analysis, log2-SII (OR = 2.22, 95% CI: 1.13–4.37, *p* = 0.021) and log2-AISI (OR = 3.27, 95% CI: 1.83–5.85, *p* < 0.001) emerged as independent risk factors for PCa. The AISI-based model achieved superior diagnostic performance with an AUC of 0.878 (95% CI: 0.825–0.931) compared with the SII-based model (AUC = 0.855, 95% CI: 0.794–0.915). DCA showed greater clinical net benefit for the AISI model across a wide range of threshold probabilities.

**Conclusion:**

Blood inflammatory markers, particularly AISI, in combination with conventional biomarkers, offer enhanced diagnostic accuracy for differentiating BPH from PCa, representing a non-invasive and cost-effective approach for clinical decision-making.

## 1 Introduction

Prostate cancer (PCa) is the second most frequently diagnosed malignancy among men worldwide and is a leading cause of cancer-related mortality in the male population. With the accelerating aging of populations and the widespread implementation of prostate-specific antigen (PSA) screening, its incidence has shown a marked upward trend. Epidemiological data indicate that approximately 1.45 million new cases and 375,000 deaths from PCa were recorded globally in 2020 (1). Beyond causing physical and psychological distress, PCa places a substantial burden on healthcare systems, with annual treatment costs and indirect social expenditures reaching hundreds of billions of dollars (2).

Benign prostatic hyperplasia (BPH) shares notable clinical similarities with PCa and is a common non-malignant prostatic disorder primarily affecting middle-aged and older men. Although both BPH and PCa predominantly occur in older males and present with similar clinical manifestations such as urinary dysfunction and mild PSA elevation, they differ fundamentally in their underlying pathology, therapeutic approaches, and prognosis (3). Accurate differentiation between BPH and PCa is of critical clinical importance, as it not only helps prevent overtreatment in BPH but also ensures timely and appropriate therapy for PCa.

Currently, transrectal ultrasound-guided prostate biopsy (TRUS-Bx) remains the gold standard for PCa diagnosis. However, this approach has notable limitations: its invasive nature may lead to complications such as bleeding, infection, and pain; moreover, biopsy procedures carry a 20–30% false-negative rate, with up to 25% of patients requiring repeat or multiple biopsies for definitive diagnosis, thereby increasing patient discomfort, healthcare costs, and complication risks (4). Research demonstrated that even with advanced biopsy techniques, a considerable proportion of PCa cases may be missed during initial procedures (5). Thus, there is an urgent clinical need to develop efficient, non-invasive diagnostic methods capable of accurately distinguishing BPH from PCa.

In this study, the term “tumor markers” specifically refers to PSA and its directly derived forms. PSA is the most widely used screening marker for PCa; however, its specificity remains limited. Research indicated that when PSA levels fall within the gray zone (4–10 ng/mL), diagnostic specificity for PCa is only 25–40%, resulting in numerous unnecessary biopsies (6). Additionally, PSA-derived indicators, including the free-to-total PSA ratio (F/TPSA) and PSA density (PSAD), have improved diagnostic performance but do not fully overcome PSA’s specificity limitations (7). Therefore, our study focused on the combination of TPSA and FPSA with novel inflammatory indices, rather than on other commercially available biomarker panels (e.g., PHI, 4Kscore).

Recent evidence increasingly indicates that inflammatory responses play critical roles in the development and progression of prostatic diseases. Studies demonstrate that inflammation is a common underlying factor in both BPH and PCa, though distinct inflammatory patterns and molecular mechanisms are observed between these conditions (8). Inflammation in BPH primarily functions through promoting tissue repair and cellular proliferation, whereas chronic inflammation in PCa facilitates carcinogenesis through mechanisms including reactive oxygen and nitrogen species production, DNA damage promotion, and oncogene activation. Sfanos et al. reported that approximately 70–80% of PCa patients exhibit varying degrees of chronic inflammatory responses, a proportion significantly higher than in BPH patients (9).

Among the many indicators reflecting systemic inflammatory status, the Systemic Immune-Inflammation Index (SII) and the Aggregate Index of Systemic Inflammation (AISI) have attracted considerable attention because of their comprehensive nature and ease of use. The primary advantages of SII and AISI as inflammatory markers stem from their derivation from routine complete blood count tests, requiring no additional examinations, which makes them economically practical and widely applicable in clinical practice. Furthermore, these indices demonstrate relative stability, being less susceptible to short-term fluctuations, and can reflect long-term inflammatory status, making them particularly suitable for evaluating chronic conditions such as prostatic diseases (10). Extensive research has confirmed the close associations of SII and AISI with the occurrence, progression, and prognosis of various diseases. In lung cancer, SII acts not only as an independent prognostic factor for non–small cell lung cancer but also helps distinguish malignant from benign pulmonary nodules (11). In hepatocellular carcinoma, elevated SII levels are strongly associated with postoperative recurrence and overall survival, serving as an independent risk factor for recurrence (12). Beyond malignant tumors, SII and AISI also show considerable value in cardiovascular diseases. SII is positively correlated with coronary artery disease severity and major adverse cardiovascular event risk, surpassing traditional single inflammatory markers in assessing disease severity and predicting prognosis (13). Yang et al. confirmed that AISI is a powerful predictor of all-cause and cardiovascular mortality in female cancer patients (14).

However, despite extensive research on the diagnostic and prognostic value of SII and AISI across various diseases, few studies have investigated their utility in differentiating BPH from PCa. Some evidence suggests that PCa patients have significantly higher SII levels than BPH patients, but these studies involved small sample sizes (*n* = 93) and lacked external validation (15). Fan et al. explored the prognostic value of inflammatory models based on NLR and PLR in PCa patients but did not systematically assess the diagnostic potential of SII and AISI (16). Given the ongoing clinical challenge of distinguishing BPH from PCa and the limitations of existing research, this study aims to systematically evaluate the diagnostic performance of blood inflammatory factors in differentiating these conditions, develop highly sensitive and specific diagnostic models, and provide more precise differential diagnostic strategies for prostatic diseases in clinical practice while reducing healthcare costs.

## 2 Methods

### 2.1 Study population

**Figure 1:**
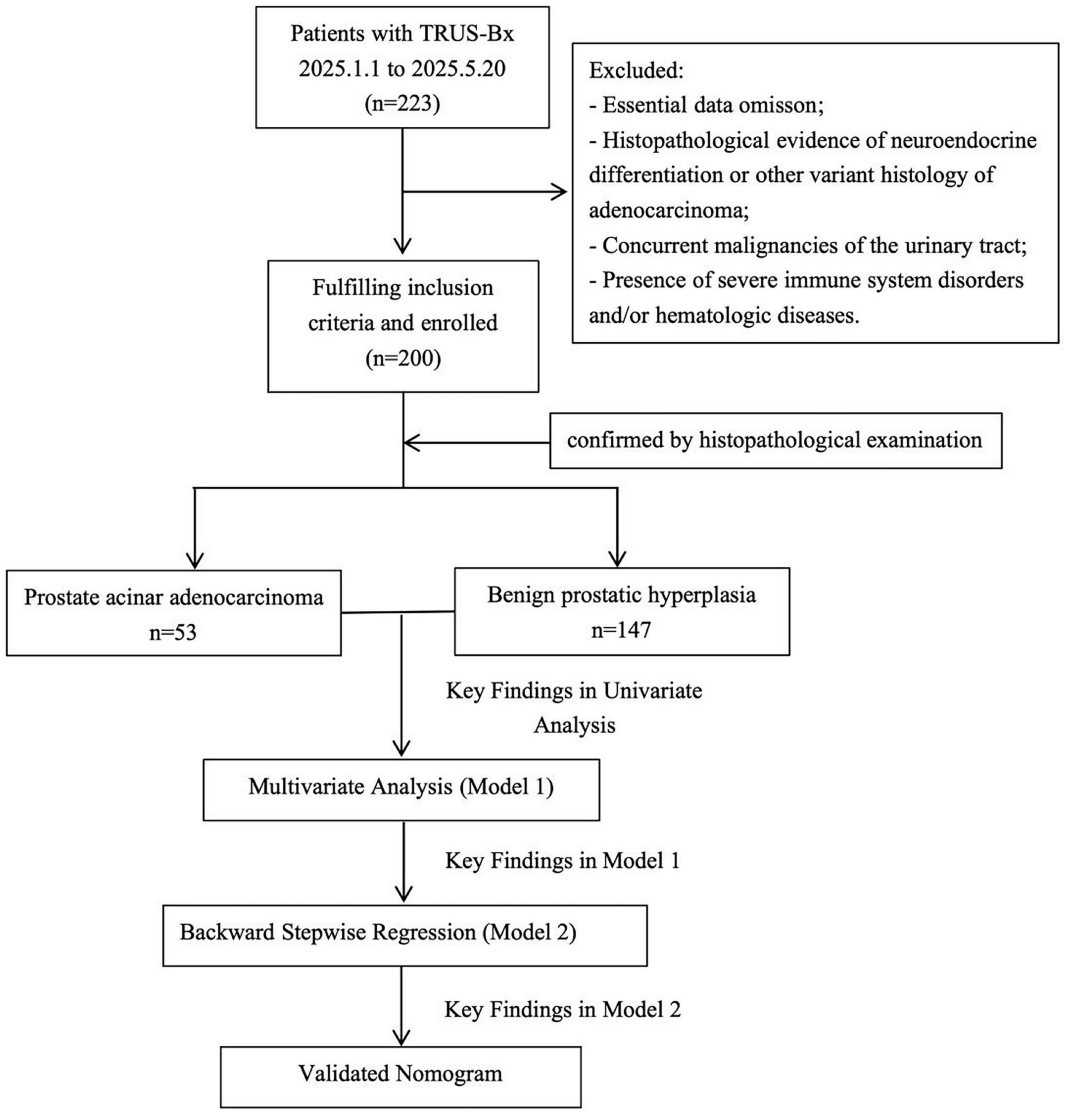
Participant selection flowchart.

This retrospective cohort study included patients who underwent ultrasound-guided prostate tissue biopsy or surgical resection at Peking University Third Hospital between January and May 2025. Figure 1 illustrated the selection process for our study. Patients were divided into two groups: those with PCa group and those with BPH group. Inclusion criteria: (1) Male patients aged 18–85 years; (2) Pathologically confirmed diagnosis of prostate acinar adenocarcinoma or BPH; and (3) Complete clinical data and laboratory parameters available. Exclusion criteria: (1) Concurrent other urological malignancies; (2) Pathological types with neuroendocrine differentiation or other special adenocarcinoma types; (3) History of psychiatric disorders; (4) Presence of severe immune system diseases and hematological disorders; and (5) Abnormal hepatic or renal function. To ensure the reliability of the results, this study adopted rigorous methods, excluded unclear, unknown, or missing data. As shown in Table 1, the final dataset included only patients meeting these stringent standards to ensure reliable analysis. This retrospective study adhered to principles outlined in the Declaration of Helsinki and received ethical approval from the Institutional Review Board (Ethics No.: (2025) Medical Ethics Review No. (412-01)). Due to the retrospective nature of this study, the committee also approved waiver of informed consent requirements.

**Table 1:** Baseline characteristics.

Variables	Overall (*n* = 200)	BPH (*n* = 147)	PCa (*n* = 53)	*P*-value
Age, years, mean (SD)	70.58 ± 7.50	71.07 ± 7.76	69.23 ± 6.63	0.101
Height, cm, mean (SD)	170.12 ± 5.55	169.84 ± 5.75	170.87 ± 4.93	0.218
Weight, kg, mean (SD)	69.88 ± 9.48	69.27 ± 9.30	71.57 ± 9.87	0.144
BMI, kg/m^2^, mean (SD)	24.12 ± 2.84	23.99 ± 2.79	24.48 ± 2.95	0.298
Smoking status, *n* (%)				0.039
No	141 (70.50%)	110 (74.83%)	31 (58.49%)	
Yes	59 (29.50%)	37 (25.17%)	22 (41.51%)	
Alcohol user, *n* (%)				0.002
No	137 (68.50%)	110 (74.83%)	27 (50.94%)	
Yes	63 (31.50%)	37 (25.17%)	26 (49.06%)	
Hypertension, *n* (%)				0.258
No	102 (51.00%)	79 (53.74%)	23 (43.40%)	
Yes	98 (49.00%)	68 (46.26%)	30 (56.60%)	
Diabetes, *n* (%)				0.079
No	155 (77.50%)	119 (80.95%)	36 (67.92%)	
Yes	45 (22.50%)	28 (19.05%)	17 (32.08%)	
CAD, *n* (%)				0.625
No	179 (89.50%)	133 (90.48%)	46 (86.79%)	
Yes	21 (10.50%)	14 (9.52%)	7 (13.21%)	
Neut (10^9^/L)	5.77 ± 1.63	5.42 ± 1.56	6.76 ± 1.40	<0.001
Mono (10^9^/L)	0.58 ± 0.18	0.54 ± 0.15	0.71 ± 0.21	<0.001
PLT (10^9^/L)	182.60 ± 52.68	175.95 ± 47.19	201.04 ± 62.42	0.009
Lymph (10^9^/L)	1.20 ± 0.35	1.16 ± 0.29	1.34 ± 0.44	0.007
SII	921.76 ± 408.77	860.13 ± 343.54	1092.69 ± 517.28	0.003
AISI	550.13 ± 311.73	482.24 ± 280.12	738.43 ± 319.99	<0.001
log2-SII	9.72 ± 0.63	9.63 ± 0.62	9.97 ± 0.59	0.001
log2-AISI	8.87 ± 0.86	8.67 ± 0.86	9.40 ± 0.60	<0.001
T-CHO (mmol/L)	4.17 ± 0.95	4.01 ± 0.89	4.62 ± 0.96	<0.001
TG (mmol/L)	1.30 ± 0.83	1.23 ± 0.79	1.49 ± 0.93	0.072
HDL (mmol/L)	1.17 ± 0.29	1.19 ± 0.29	1.14 ± 0.29	0.284
LDL (mmol/L)	2.53 ± 0.74	2.39 ± 0.69	2.93 ± 0.76	<0.001
Glu (mmol/L)	6.05 ± 1.98	5.92 ± 1.89	6.43 ± 2.20	0.134
ALT (U/L)	19.81 ± 11.91	19.61 ± 10.97	20.36 ± 14.30	0.731
AST (U/L)	23.23 ± 7.60	23.27 ± 8.06	23.09 ± 6.24	0.870
TP (g/L)	65.87 ± 8.56	67.11 ± 8.19	62.43 ± 8.69	0.001
CysC (mg/L)	1.07 ± 0.33	1.01 ± 0.28	1.25 ± 0.41	<0.001
TBIL (umol/L)	18.44 ± 9.62	17.82 ± 9.04	20.13 ± 10.99	0.174
APTT (s)	32.01 ± 3.23	32.06 ± 3.27	31.89 ± 3.14	0.752
PT (s)	10.89 ± 0.69	10.94 ± 0.69	10.73 ± 0.66	0.049
TT (s)	14.43 ± 1.04	14.44 ± 1.05	14.40 ± 1.01	0.779
FIB (g/L)	3.20 ± 0.82	3.23 ± 0.84	3.12 ± 0.78	0.421
TPSA (ng/ml)	7.82 ± 3.76	7.08 ± 3.76	9.86 ± 2.96	<0.001
FPSA (ng/ml)	1.57 ± 1.33	1.44 ± 1.33	1.93 ± 1.29	0.018

#: BMI, body mass index; CAD, coronary artery disease; Neut, neutrophils; Mono, monocytes; PLT, platelets; Lymph, lymphocytes; SII, systemic immune-inflammation index; AISI, aggregate index of systemic inflammation; T-CHO, total cholesterol; TG, triglycerides; HDL, high density lipoprotein; LDL, low density lipoprotein; Glu, glucose; ALT, alanine aminotransferase; AST, aspartate aminotransferase; TP, total protein; CysC, cystatin c; TBIL, total bilirubin; APTT, activated partial thromboplastin time; PT, prothrombin time; TT, thrombin time; FIB, fibrinogen; TPSA, total prostate specific antigen; FPSA, free prostate specific antigen.

### 2.2 Data collection

Patient data were extracted from electronic medical records, including comprehensive demographic, lifestyle, clinical, and laboratory parameters. Demographic variables included age, gender, height, and weight. Lifestyle factors including smoking and alcohol consumption were recorded as categorical variables. Clinical data encompassed underlying conditions such as hypertension, diabetes, and coronary artery disease.

Laboratory parameters included peripheral blood complete blood count, biochemistry, coagulation studies, and prostate tumor markers. Laboratory measurements were standardized using calibrated equipment to ensure accuracy.

### 2.3 Definition of PCa and BPH

#### 2.3.1 Definition of PCa

In this study, prostate acinar adenocarcinoma was defined as malignant epithelial tumors originating from prostatic acini, confirmed by pathological examination of biopsy or surgical specimens. Prostate acinar adenocarcinoma represents the most common histological type of prostate malignancy, comprising approximately 95% of cases, characterized by glandular structure formation (including acini, small glands, fused glands, cribriform structures), with Gleason grading system and ISUP grading applicable to this type (17).

#### 2.3.2 Definition of BPH

BPH was defined as pathologically confirmed benign proliferation of prostatic stromal and epithelial components, with clinical manifestations potentially including lower urinary tract symptoms (LUTS), prostate volume enlargement, and/or bladder outlet obstruction (BOO). According to recent reviews by Kaplan, BPH represents an age-progressive disease with approximately 50% prevalence in men over 50 years and up to 90% in those over 80 years (18).

### 2.4 Definition of inflammatory factors

#### 2.4.1 Definition of SII

SII is a comprehensive inflammatory marker based on routine peripheral blood examination. First proposed by Hu et al. (19), SII is calculated as: SII = P × N/L, where P represents platelet count, N represents neutrophil count, and L represents lymphocyte count. SII comprehensively reflects systemic inflammatory responses and immune functional status, simultaneously evaluating platelet-mediated adhesion reactions, neutrophil-mediated inflammatory responses, and lymphocyte-mediated immune responses in circulating blood. Compared to single ratios such as neutrophil-to-lymphocyte ratio (NLR) or platelet-to-lymphocyte ratio (PLR), SII provides more comprehensive inflammatory microenvironment assessment.

#### 2.4.2 Definition of AISI

AISI represents a comprehensive inflammatory indicator further integrating monocyte count based on SII, calculated as: AISI = N × M × P/L, where N represents neutrophil count, M represents monocyte count, P represents platelet count, and L represents lymphocyte count (20). Monocytes play important roles in inflammatory responses, differentiating into macrophages and participating in tissue repair and immune regulation. Research by Suzuki et al. (21) confirmed that AISI, by incorporating monocytes as important inflammatory cells, provides more comprehensive systemic inflammation assessment than SII, demonstrating advantages in diagnosis and prognosis evaluation across various tumors.

### 2.5 Statistical analysis

Descriptive statistical methods were employed to summarize demographic characteristics, clinical presentations, laboratory examinations, and coagulation function data. Continuous variables were expressed as mean ± standard deviation, while categorical variables were presented as frequencies and percentages. *T*-tests were used to compare continuous variable differences between PCa and BPH groups, while chi-square tests analyzed categorical variable differences. Univariate logistic regression evaluated associations between variables and PCa risk. Variables with *p*-values <0.05 in univariate analysis were included in multivariate logistic regression models, with backward stepwise regression used to screen independent predictors of PCa. This backward stepwise method prioritized variables based on clinical relevance and statistical significance, ensuring final models retained only strong predictors, calculating odds ratios (OR) and 95% confidence intervals (CI).

Nomograms were constructed based on significant predictors from multivariate analysis for intuitive model presentation. Receiver operating characteristic (ROC) curves evaluated model performance, with area under the curve (AUC) reflecting predictive accuracy. Calibration curves assessed model calibration, while decision curve analysis (DCA) examined clinical utility.

All statistical analyses were performed using the Statistical Package for Social Sciences (SPSS) version 27.0 (including backward stepwise regression models) and R software (version 4.1.3; R Foundation for Statistical Computing, Vienna, Austria), with the significance level set at *p* < 0.05.

## 3 Results

### 3.1 Baseline characteristics

Based on predetermined inclusion and exclusion criteria, this study enrolled 200 patients. The overall population had a mean age of 70.58 ± 7.50 years, BMI of 24.12 ± 2.84 kg/m^2^, with 59 smokers (29.50%), 63 alcohol consumers (31.50%), 98 patients with hypertension (49.00%), 45 with diabetes (22.50%), and 21 with coronary heart disease (10.50%). According to pathological diagnosis, patients were divided into BPH group (*n* = 147) and PCa group (*n* = 53). Compared to the BPH group, the PCa group showed more significant differences in lifestyle-related variables, with higher smoking rates (41.51% vs. 25.17%, *p* = 0.039) and alcohol consumption rates (49.06% vs. 25.17%, *p* = 0.002). No significant differences were observed between groups in baseline characteristics such as age, height, weight, and BMI. Various inflammatory markers (including SII, AISI, log2-SII, and log2-AISI) were significantly higher in the acinar adenocarcinoma group compared to the benign group (*p* < 0.05). Details are shown in Table 1.

### 3.2 Univariate logistic regression analysis

Table 2 demonstrated that univariate logistic regression analysis identified multiple factors significantly associated with prostate acinar adenocarcinoma occurrence. log2-SII and log2-AISI showed significant positive correlations with increased acinar adenocarcinoma risk. Additionally, smoking and alcohol consumption behaviors, lipid-related indicators (total cholesterol and low-density lipoprotein), total protein, cystatin C, and prostate-specific antigen indicators (TPSA and FPSA) were also associated with acinar adenocarcinoma occurrence (all *p* < 0.05). In contrast, age, BMI, hypertension, diabetes, coronary heart disease, and most routine biochemical and coagulation indicators showed no significant associations (all *p* ≥ 0.05).

**Table 2:** Univariate logistic regression analysis.

Variables	OR (95%CI)	*P*-value
Age, years, mean (SD)	0.97 (0.93, 1.01)	0.128
BMI	1.06 (0.95, 1.19)	0.283
BMI, kg/m^2^, mean (SD)		
No	Reference	
Yes	2.10 (1.08, 4.09)	0.030
Alcohol user, *n* (%)		
No	Reference	
Yes	2.84 (1.47, 5.52)	0.002
Hypertension, *n* (%)		
No	Reference	
Yes	1.51 (0.80, 2.87)	0.202
Diabetes, *n* (%)		
No	Reference	
Yes	2.00 (0.97, 4.07)	0.060
CAD, *n* (%)		
No	Reference	
Yes	1.46 (0.52, 3.77)	0.460
log2-SII	2.62 (1.48, 4.64)	0.001
log2-AISI	3.47 (2.12, 5.68)	<0.001
T-CHO (mmol/L)	2.12 (1.45, 3.09)	<0.001
TG (mmol/L)	1.41 (0.98, 2.02)	0.065
HDL (mmol/L)	0.54 (0.18, 1.66)	0.286
LDL (mmol/L)	2.86 (1.76, 4.63)	<0.001
Glu (mmol/L)	1.13 (0.97, 1.31)	0.111
ALT (U/L)	1.01 (0.98, 1.03)	0.696
AST (U/L)	1.00 (0.96, 1.04)	0.884
TP (g/L)	0.94 (0.90, 0.97)	0.001
CysC (mg/L)	7.49 (2.86, 19.6)	<0.001
TBIL (umol/L)	1.02 (0.99, 1.06)	0.140
APTT (s)	0.98 (0.89, 1.09)	0.755
PT (s)	0.61 (0.37, 1.01)	0.054
TT (s)	0.96 (0.71, 1.30)	0.782
FIB (g/L)	0.85 (0.57, 1.28)	0.435
TPSA (ng/ml)	1.23 (1.12, 1.34)	<0.001
FPSA (ng/ml)	1.29 (1.03, 1.61)	0.024

#: BMI, body mass index; CAD, coronary artery disease; SII, systemic immune-inflammation index; AISI, aggregate index of systemic inflammation; T-CHO, total cholesterol; TG, triglycerides; HDL, high density lipoprotein; LDL, low density lipoprotein; Glu, glucose; ALT, alanine aminotransferase; AST, aspartate aminotransferase; TP, total protein; CysC, cystatin c; TBIL, total bilirubin; APTT, activated partial thromboplastin time; PT, prothrombin time; TT, thrombin time; FIB, fibrinogen; TPSA, total prostate specific antigen; FPSA, free prostate specific antigen.

### 3.3 Multiple logistic regression models

After including significant univariate variables in multivariate models, we constructed two separate multivariate logistic regression models and used backward elimination for variable selection and model optimization. Both models considered covariates including smoking, alcohol consumption, T-CHO, LDL, TP, CysC, TPSA, and FPSA. In the SII model (Table 3), Model 1 demonstrated a likelihood ratio chi-square value of 75.145 (*p* < 0.001), indicating good overall model fit, and table of the log2-SII multivariate model parameters were shown in Supplementary Table S1. Model 1 of log2-SII multivariate model showed log2-SII was a risk factor of PCa (OR = 2.29, 95%CI: 1.16–4.52, *p* = 0.017). The optimized model obtained through backward elimination (Model 2) showed a likelihood ratio chi-square value of 73.211 (*p* < 0.001), with log2-SII maintaining significant positive correlation with PCa risk (OR = 2.22, 95%CI: 1.13–4.37, *p* = 0.021), and table of the optimized log2-SII multivariate model parameters were shown in Supplementary Table S2. In the AISI model (Table 3), Model 1 demonstrated a likelihood ratio chi-square value of 86.991 (*p* < 0.001), with log2-AISI significantly associated with PCa risk (OR = 3.16, 95%CI: 1.76–5.68, *p* < 0.001), and table of the log2-AISI multivariate model parameters were shown in Supplementary Table S3. The optimized model obtained through backward elimination (Model 2) showed a likelihood ratio chi-square value of 84.906 (*p* < 0.001), with logAISI maintaining stable significance (OR = 3.27, 95%CI: 1.83–5.85, *p* < 0.001), and table of the optimized log2-AISI multivariate model parameters were shown in Supplementary Table S4. A VIF value more than 10 or a TOL less than 0.1 indicate multicollinearity. In addition, there were no multicollinearity among the variables in the optimized log2-SII model (Supplementary Table S5) and the optimized log2-AISI model (Supplementary Table S6).

**Table 3:** Multiple logistic regression models of log2-SII (A), log2-AISI (B).

(A) log2-SII				
Likelihood ratio chi-square	Model 1		Model 2	
	75.145	<0.001	73.211	<0.001
Variables	OR (95%CI)	*P*	OR (95%CI)	*P*
log2-SII	2.29 (1.16, 4.52)	0.017	2.22 (1.13, 4.37)	0.021
Smoking status (Yes)	1.68 (0.57, 4.90)	0.346		
Alcohol user (Yes)	2.03 (0.70, 5.90)	0.193	2.73 (1.19, 6.30)	0.018
T-CHO (mmol/L)	1.35 (0.73, 2.49)	0.340		
LDL (mmol/L)	2.47 (1.08, 5.66)	0.033	3.15 (1.72, 5.77)	<0.001
TP (g/L)	0.95 (0.91, 1.00)	0.059	0.95 (0.91, 1.00)	0.048
CysC (mg/L)	8.18 (2.32, 28.85)	0.001	7.62 (2.26, 25.76)	0.001
TPSA (ng/ml)	1.19 (1.05, 1.34)	0.005	1.19 (1.06, 1.32)	0.002
FPSA (ng/ml)	1.01 (0.73, 1.40)	0.955		
#: SII, systemic immune-inflammation index; T-CHO, total cholesterol; LDL, low density lipoprotein; Glu, glucose; TP, total protein; CysC, cystatin c; TPSA, total prostate specific antigen; FPSA, free prostate specific antigen.				

(B) log2-AISI				
Likelihood ratio chi-square	Model 1		Model 2	
	86.991	<0.001	84.906	<0.001
Variables	OR (95%CI)	*P*	OR (95%CI)	*P*
log2-AISI	3.16 (1.76, 5.68)	<0.001	3.27 (1.83, 5.85)	<0.001
Smoking status (Yes)	1.77 (0.58, 5.35)	0.314		
Alcohol user (Yes)	1.62 (0.53, 4.96)	0.398		
T-CHO (mmol/L)	1.44 (0.76, 2.76)	0.265		
LDL (mmol/L)	2.20 (0.92, 5.28)	0.077	3.01 (1.64, 5.53)	<0.001
TP (g/L)	0.95 (0.90, 1.00)	0.054	0.95 (0.90, 1.00)	0.043
CysC (mg/L)	7.89 (2.14, 29.16)	0.002	8.08 (2.37, 27.58)	0.001
TPSA (ng/ml)	1.19 (1.05, 1.34)	0.006	1.18 (1.06, 1.32)	0.002
FPSA (ng/ml)	1.07 (0.77, 1.49)	0.673		

#: AISI, aggregate index of systemic inflammation; T-CHO, total cholesterol; LDL, low density lipoprotein; TP, total protein; CysC, cystatin c; TPSA, total prostate specific antigen; FPSA, free prostate specific antigen.

### 3.4 Nomogram for PCa prediction

**Figure 2:**
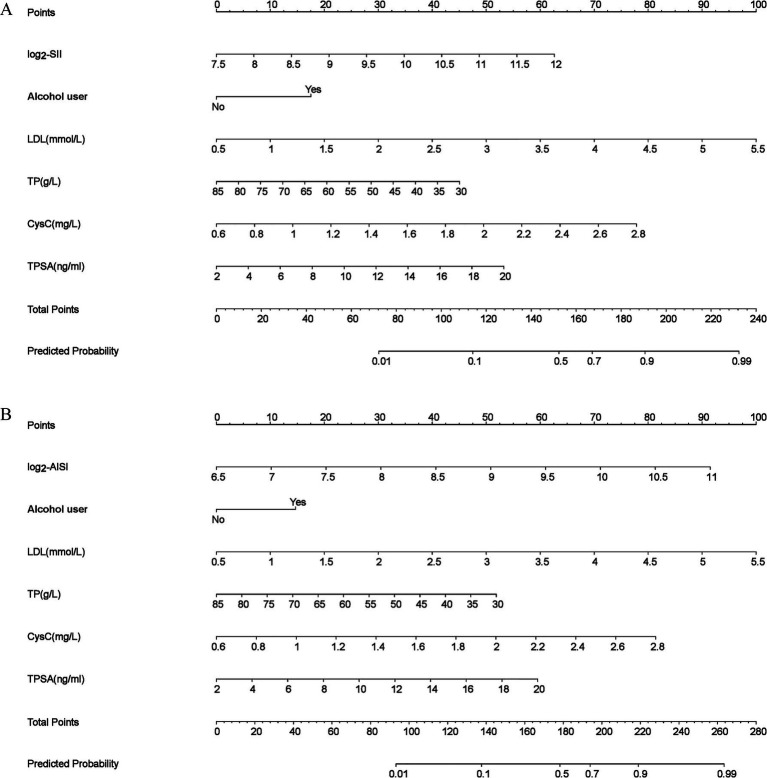
Nomogram models **(a)** log2-SII; **(b)** log2-AISI. The odds ratio (OR) > 1.00, indicating that the variable is associated with an increased risk of PCa; scores were obtained from each scale according to patient-specific indicators, with total scores corresponding to predicted probabilities after summation. Higher total scores indicated greater PCa risk.

Based on the optimized results (Model 2), SII (Figure 2A) and AISI (Figure 2B) nomograms were constructed, respectively. The results showed higher SII, higher AISI, alcohol user, higher LDL, lower TP, higher CysC and higher TPSA were risk factors of PCa. When using the nomogram to predict PCa, doctors can place on the corresponding axes based on the patient’s risk factors. The assigned scores from each factor were summed to obtain a total score. A higher total score indicated a greater risk of PCa.

### 3.5 Calibration analysis

**Figure 3:**
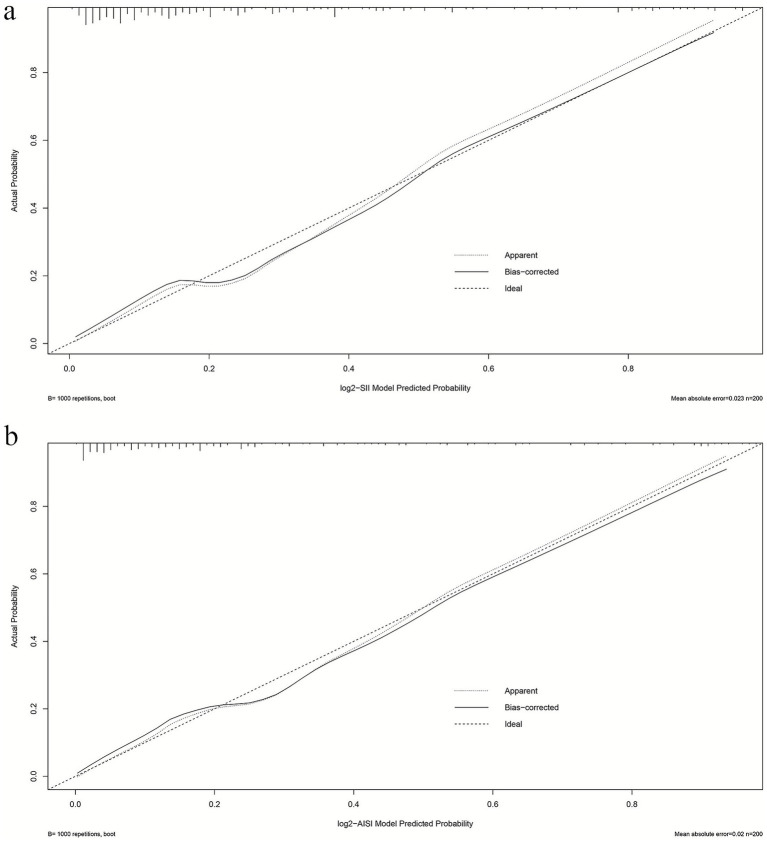
Calibration profiles of multiple logistic regression models **(a)** log_2_-SII; **(b)** log_2_-AISI. The calibration curve was generated using 1,000 bootstrap repetitions. The diagonal dashed line represents the ideal case of perfect prediction, while the solid line indicates the actual performance of our model. Closer agreement between the two lines signifies better predictive accuracy.

Calibration performance of both Model 2 frameworks was comparatively evaluated. The slope and Brier score were used to numerically assess the accuracy of model predictions; a slope closer to 1 indicates better predictive accuracy, while a Brier score of less than 0.25 indicates that the model’s predictions are reasonably accurate. The SII model’s calibration slope was 1.000 and Brier score was 0.122 (Supplementary Figure S1); the AISI model’s calibration slope was 1.000 and Brier score was 0.114 (Supplementary Figure S2). The SII model’s calibration curve (Figure 3A) maintained good concordance with the ideal line, with mean absolute error (MAE) of 0.023, indicating acceptable overall calibration. The AISI model’s calibration curve (Figure 3B) demonstrated high consistency between predicted and actual probabilities, with bias-corrected curves nearly overlapping the ideal line and MAE of 0.020, suggesting high calibration precision. The AISI model exhibited lower prediction error and calibration characteristics closer to the ideal line, with superior calibration accuracy compared to the SII model.

### 3.6 ROC analysis and model comparison

**Figure 4:**
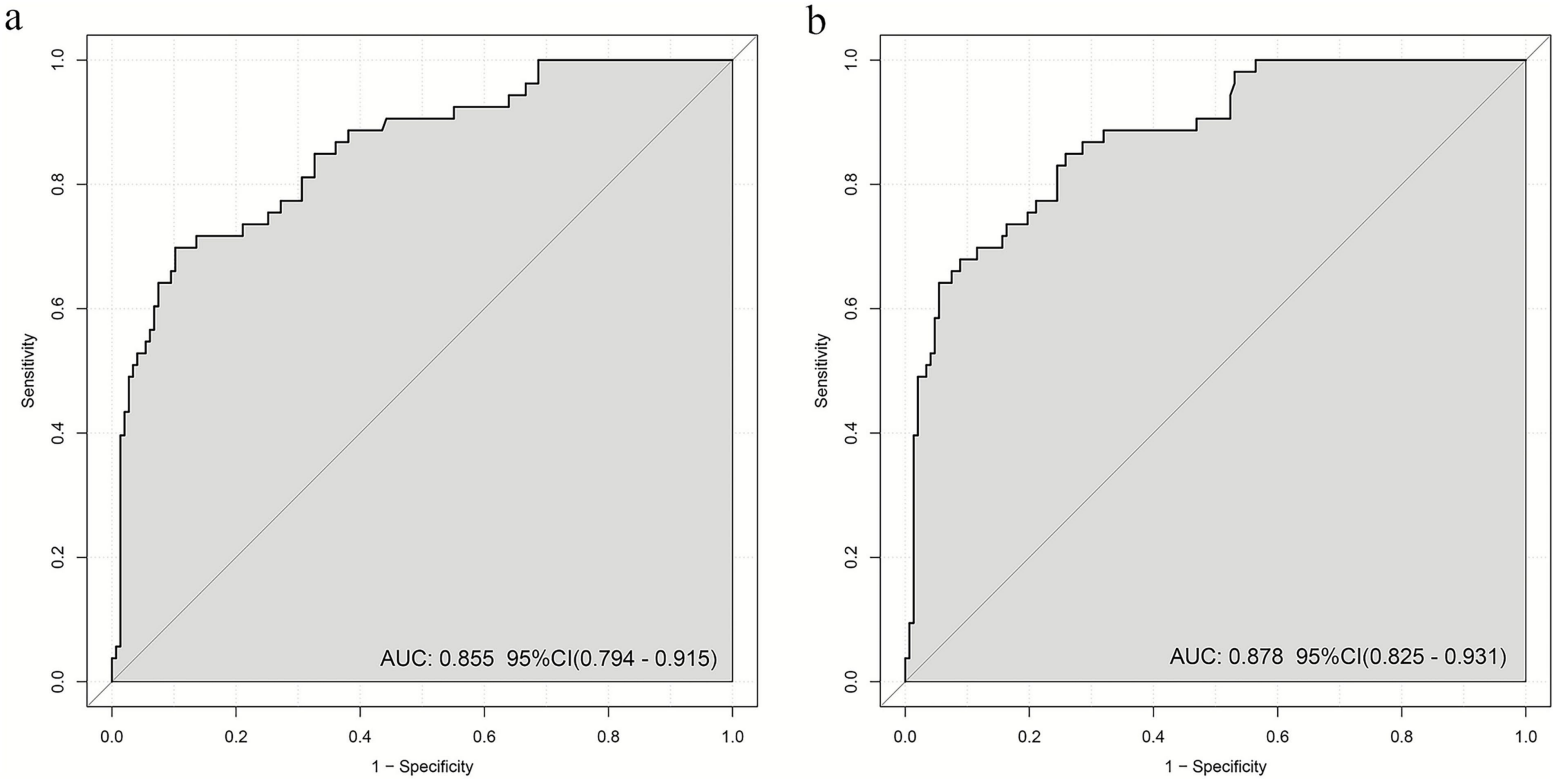
AUC values of multiple logistic regression models: **(a)** log_2_-SII; **(b)** log_2_-AISI. AUC over 0.7 indicating that the model possesses a relatively good discriminatory ability and accuracy.

Based on the two optimized models, ROC curves were generated to evaluate discriminative performance. The SII model (Figure 4A) achieved an AUC of 0.855 (95%CI: 0.794–0.915), the optimism-corrected AUC was 0.832; while the AISI model (Figure 4B) achieved an AUC of 0.878 (95%CI: 0.825–0.931), the optimism-corrected AUC was 0.858, indicating significantly higher AUC for the AISI model compared to the SII model (DeLong’s test showed z = 1.976, ΔAUC = 0.023, *p* = 0.039).

### 3.7 DCA

**Figure 5:**
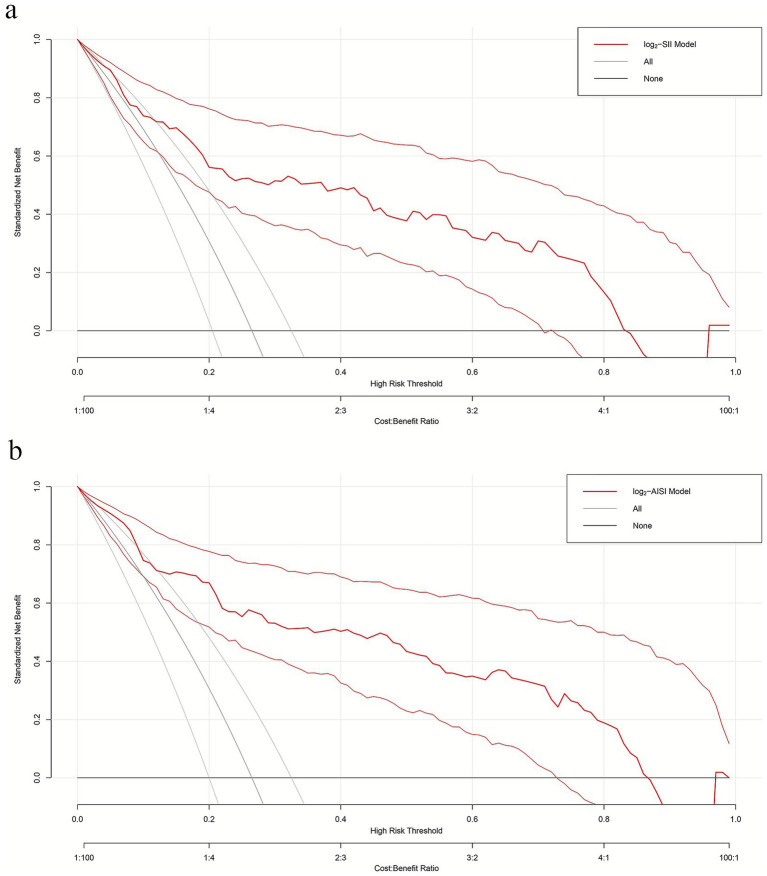
Decision curves of multiple logistic regression models **(a)** log2-SII; **(b)** log2-AISI. Nomograms offered a greater net benefit compared to both the “treat-all” and “treat-none” approaches over a threshold probability range from 10 to 80%, confirming its clinical utility in routine practice.

To evaluate model net benefit across different clinical threshold probabilities, DCA was performed for both log2-SII (Figure 5A) and log2-AISI (Figure 5B) optimized models. Within commonly used threshold probability ranges (approximately 10–80%), both models demonstrated standardized net benefits significantly higher than “treat all” and “treat none” strategies, indicating practical clinical application value.

### 3.8 Relationship between SII, AISI, and PCa

In the overall patients, univariate logistic regression results (Model 1) showed a significant positive association between log2-SII (OR: 2.62, 95% CI: 1.48, 4.64, *p* < 0.001), log2-AISI (OR: 3.47, 95% CI: 2.12, 5.68, *p* < 0.001) and PCa risk. After adjusting for age, BMI, smoking status and alcohol user in Model 2, this positive association remained (log2-SII, OR: 2.50, 95% CI: 1.40, 4.44, *p* = 0.002; log2-AISI, OR: 3.20, 95% CI: 1.94, 5.28, *p* < 0.001). After adjusting for all covariates in Model 3, log2-SII (OR: 2.25, 95% CI: 1.20, 4.23, *p* = 0.011), log2-AISI (OR: 3.29, 95% CI: 1.88, 5.77, *p* < 0.001) remained significantly associated with PCa risk. Both log2-SII and log2-AISI remained independent risk factors for PCa after multivariate adjustment. Details were shown in Table 4.

**Table 4:** Relationship between SII, AISI and PCa risk using logistic regression analyses.

Variables	Model 1		Model 2		Model 3	
	OR (95% CI)	*P*	OR (95% CI)	*P*	OR (95% CI)	*P*
log2-SII	2.62 (1.48 ~ 4.64)	<0.001	2.50 (1.40 ~ 4.44)	0.002	2.25 (1.20 ~ 4.23)	0.011
log2-AISI	3.47 (2.12 ~ 5.68)	<0.001	3.20 (1.94 ~ 5.28)	<0.001	3.29 (1.88 ~ 5.77)	<0.001

OR, odds ratio; CI, Confidence interval.

Model 1: No covariates adjusted.

Model 2: Adjusted for age, BMI, smoking status and alcohol user.

Model 3: Adjusted for age, BMI, smoking status, alcohol user, hypertension, diabetes, CAD, T-CHO, HDL, TP, CysC, TPSA and FPSA.

### 3.9 Relationship between SII, AISI and Gleason score

**Figure 6:**
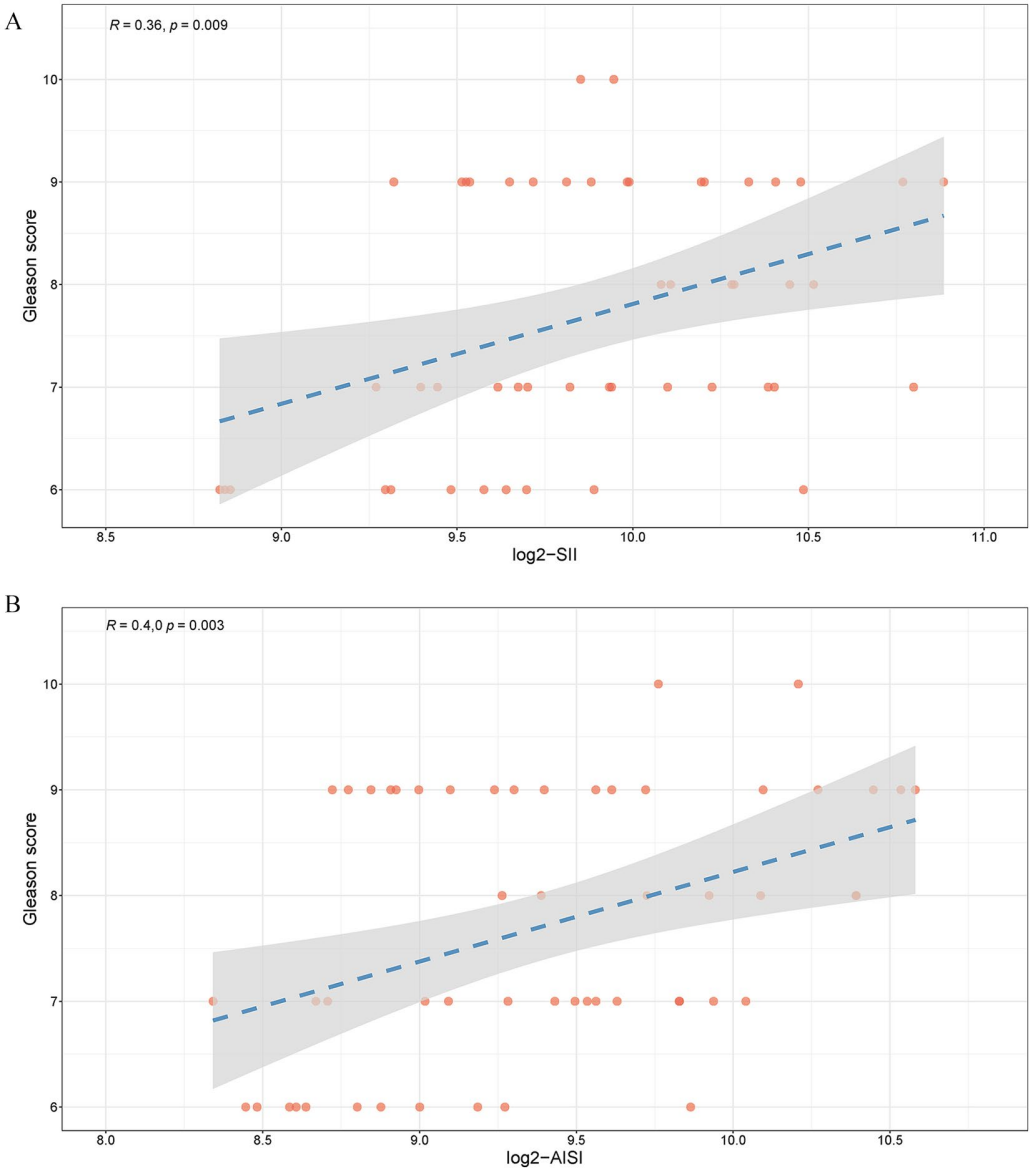
Relationship between SII, AISI and Gleason score **(a)** log_2_-SII; **(b)** log_2_-AISI. A value of *R* ranging from 0 to 1, with a *p*-value <0.05, indicates a positive correlation.

To elucidate the correlation between SII, AISI and PCa histopathological grade, Spearman’s analysis was employed to assess the association between SII, AISI and the Gleason score. The results demonstrated a significant positive correlation between SII (Figure 6A, R = 0.36, *p* = 0.009), AISI (Figure 6B, R = 0.40, *p* = 0.003) and the Gleason score.

### 3.10 Linear association between predictive factors and PCa

We used restricted cubic spline (RCS) analysis to better demonstrate the relationship between predictive factors (log2-SII, log2-AISI, LDL, TP, CysC, TPSA) and PCa (Supplementary Figure S3A-F), and observed significant linear correlations between the predictors and PCa. We conducted threshold effect analysis and found inflection points (log2-SII was 9.73, log2-AISI was 8.91, LDL was 2.43, TP was 67.10, CysC was 0.96, TPSA was 6.82). Observations indicated that when log2-SII, log2-AISI, LDL, CysC, TPSA was below the inflection point, the risk of PCa was lower, when log2-SII, log2-AISI, LDL, CysC, TPSA exceeded the inflection point, the risk increases rapidly. However, PCa risk remained high below this threshold but reduced significantly when TP exceeded 67.10.

## 4 Discussion

The occurrence and development of PCa arise from multiple interacting factors. Previous research has clearly established the contributions of age, race, family history, hormonal levels, and lifestyle factors, while chronic inflammation appears to span the entire carcinogenic process, playing key roles in tumor initiation and progression (22–24). Inflammation can reshape the local prostatic microenvironment through pathways including oxidative stress promotion, DNA damage and repair imbalance, immune evasion, cytokine cascades, and angiogenesis, thereby driving tumor cell proliferation, invasion, and metastasis (25, 26). Based on recent evidence, composite inflammatory indices may serve as powerful tools for tumor prognosis and risk stratification. In PCa and various solid tumors, these indices consistently correlate with adverse outcomes, demonstrating strong cross-tumor applicability and generalizability (27–29).

Our study found SII and AISI were independent risk factors of PCa, AUC of the optimized SII model was 0.855, and the optimized AISI model was 0.878. Compared to established biomarkers, AUC of PSA alone for predicting PCa risk was 0.615 (30). In the GÖTEBORG-2 screening trial, the 4Kscore Test achieved an AUC of 0.84 (95% CI: 0.79–0.89) for detecting intermediate to high grade PCa (31), while recent studies reported that the Prostate Health Index (PHI) demonstrated AUC values ranging from 0.81 to 0.884 for clinically significant PCa detection (32, 33). Our model demonstrated comparable superior diagnostic performance relative to PHI and the 4Kscore Test. In addition, we found AISI demonstrated a superior predictive ability compared to SII, as AISI incorporates monocytes, it can more comprehensively capture myeloid-driven immune suppression and inflammatory signals.

Accumulating evidence suggests that systemic inflammation may contribute to PCa through several interconnected molecular pathways (34, 35). Systemic inflammatory responses can activate key signaling cascades, including NF-κB and STAT3, which have been associated with sustained production of pro-inflammatory cytokines (TNF-*α*, IL-6, IL-1β) that may facilitate tumor cell proliferation, survival, angiogenesis, and immune evasion within the prostate tumor microenvironment. Additionally, inflammation-mediated oxidative stress has been reported to generate reactive oxygen species that may induce DNA damage and genomic instability in prostatic epithelial cells, while inflammatory mediators have been implicated in promoting epithelial-to-mesenchymal transition and establishing an immunosuppressive microenvironment that potentially supports cancer progression. Neutrophils act as core effector cells in acute inflammation and can adopt protumor phenotypes within the tumor microenvironment. By releasing elastase, myeloperoxidase, reactive oxygen species, and neutrophil extracellular traps (NETs), they promote tumor cell invasion, epithelial–mesenchymal transition (EMT), and distant metastasis while suppressing T-cell effector functions (36). Lymphocytes play a crucial role in anti-tumor immunity, with CD8^+^ cytotoxic T cells and natural killer (NK) cells directly eliminating tumor cells through the release of cytotoxic molecules such as perforin and granzyme, as well as cytokines including interferon-*γ*. Chronic inflammation-induced immunosuppressive microenvironments promote the depletion and functional exhaustion of circulating and tumor-infiltrating lymphocytes, contributing to increased tumorigenesis risk and unfavorable clinical outcomes (37). Tumor-activated platelets facilitate metastasis by shielding circulating tumor cells from immune surveillance and secreting growth factors (VEGF, PDGF, TGF-*β*) that promote epithelial-mesenchymal transition, angiogenesis, and metastatic colonization (38). Elevated platelet counts or activation states are associated with adverse outcomes across multiple cancer types, which helps explain the positive correlation between platelet inclusion in indices and PCa risk. The monocyte/macrophage axis plays a pivotal role in chronic inflammation and tumor immune evasion. After entering tumor microenvironments, monocytes differentiate into tumor-associated macrophages. When polarized toward M2-like phenotypes, they secrete IL-10, TGF-β, and matrix metalloproteinases, thereby suppressing anti-tumor immunity and promoting invasion and metastasis (39).

Beyond inflammation-related indicators, this study also identified several secondary yet meaningful covariates. Alcohol consumption was positively associated with PCa risk, possibly due to acetaldehyde production during alcohol metabolism, increased oxidative stress, alterations of the endocrine axis, and upregulation of pro-inflammatory cytokines (40). However, previous literature on alcohol–PCa relationships remains inconsistent, highlighting the need for more refined dose- and type-specific analyses. Elevated LDL was likewise correlated with PCa risk, with proposed mechanisms including cholesterol serving as a membrane synthesis and steroid hormone precursor that promotes tumor cell growth, and cholesterol-enriched microdomains affecting receptor signal transduction while inducing inflammatory responses and oxidative stress (41). CysC levels were significantly associated with PCa risk. Beyond reflecting glomerular filtration, CysC participates in regulating caspase pathways, extracellular matrix degradation, and tumor cell migration, indicating that it may serve as a biological link within the “renal function–inflammation–tumor” interaction network (42). TP showed marginal significance in the models, possibly reflecting indirect effects of nutritional–inflammatory status on immune function, although its effect size was small and clinical interpretation should be approached with caution.

However, this study had several limitations. First, this study only analyzed the correlation between SII, AISI, and PCa, and cannot establish a causal relationship. Second, as a single-center study without external validation, the generalizability of our findings was limited. Third, the relatively limited sample size may affect the accuracy of the conclusions. Fourth, a notable imbalance existed in the sample sizes between the study groups. Although this distribution reflected the real-world clinical prior probability in a biopsy population and enhanced the practical relevance of our findings, it could potentially introduce bias and affect model calibration. Fifth, the relatively short participant enrollment period was a notable limitation. A brief enrollment window may not adequately represent patient characteristics across the entire year, as certain relevant biomarkers (e.g., inflammatory markers) could exhibit seasonal variations. Sixth, the analysis included a restricted set of variables and did not incorporate tumor markers such as CA125, CA153, and CA199, which could potentially influence the accuracy of disease classification. Seventh, although multiple confounding factors were adjusted for in the statistical analyses, the absence of important variables such as TNF-*α*, IL-6, and CRP may have introduced residual confounding. Based on the above limitations, future research can be pursued in the following areas. First, large scale prospective cohort studies should be conducted to clarify the temporal relationship between SII, AISI, and the incidence and progression of PCa. Second, the predictive ability of SII and AISI for PCa requires further validation in multi-center studies and across diverse ethnic populations, alongside the exploration of appropriate reference ranges. Third, integrating more precise diagnostic tools for PCa could enhance the accuracy and reliability of the findings. Fourth, conducting basic research to elucidate the specific roles of SII and AISI in the pathogenesis of PCa. Finally, developing risk models that combine SII and AISI with other biomarkers or imaging features may improve the accuracy of PCa risk assessment.

## Data Availability

The original contributions presented in the study are included in the article/Supplementary material, further inquiries can be directed to the corresponding authors.
