# Supplementary material for: Blood inflammatory markers combined with tumor markers for differentiating benign prostatic hyperplasia from prostate cancer
Source: Front Med (Lausanne). 2026 Feb 4;13:1730818. doi: 10.3389/fmed.2026.1730818 (PMC12913570; doi:10.3389/fmed.2026.1730818)
Supplement: Supplementary file 1 [file Supplementary_file_1.docx]

**Table S1 Table of the log2-SII multivariate model parameters**

| **Variable** | ***β*** | ***S.D*** | ***Wald* χ²** | **OR(95%CI)** | **P-value** |
| --- | --- | --- | --- | --- | --- |
| Intercept | -13.887 | 3.984 | 12.149 | - | <0.001 |
| log2-SII | 0.827 | 0.348 | 5.653 | 2.285 (1.156 - 4.517) | 0.017 |
| Smoking status(Yes) | 0.516 | 0.547 | 0.890 | 1.675 (0.573 - 4.895) | 0.346 |
| Alcohol user(Yes) | 0.709 | 0.544 | 1.696 | 2.031 (0.699 - 5.902) | 0.193 |
| T-CHO(mmol/L) | 0.299 | 0.314 | 0.911 | 1.349 (0.730 - 2.494) | 0.340 |
| LDL(mmol/L) | 0.904 | 0.424 | 4.556 | 2.469 (1.077 - 5.663) | 0.033 |
| TP(g/L) | -0.048 | 0.025 | 3.563 | 0.953 (0.907 - 1.002) | 0.059 |
| CysC(mg/L) | 2.102 | 0.643 | 10.680 | 8.180 (2.319 - 28.849) | 0.001 |
| TPSA(ng/ml) | 0.170 | 0.061 | 7.730 | 1.186 (1.052 - 1.337) | 0.005 |
| FPSA(ng/ml) | 0.009 | 0.168 | 0.003 | 1.009 (0.727 - 1.402) | 0.955 |

**Table S2 Table of the optimized log2-SII multivariate model parameters**

| **Variable** | ***β*** | ***S.D*** | ***Wald χ²*** | **OR(95%CI)** | **P-value** |
| --- | --- | --- | --- | --- | --- |
| Intercept | -12.872 | 3.876 | 11.026 | - | 0.001 |
| log2-SII | 0.799 | 0.345 | 5.358 | 2.223 (1.130 - 4.374) | 0.021 |
| Alcohol user(Yes) | 1.005 | 0.426 | 5.566 | 2.733 (1.185 - 6.298) | 0.018 |
| LDL(mmol/L) | 1.148 | 0.309 | 13.826 | 3.152 (1.721 - 5.774) | <0.001 |
| TP(g/L) | -0.047 | 0.025 | 3.580 | 0.954 (0.909 - 1.000) | 0.048 |
| CysC(mg/L) | 2.031 | 0.621 | 10.683 | 7.622 (2.255 - 25.764) | 0.001 |
| TPSA(ng/ml) | 0.170 | 0.055 | 9.430 | 1.185 (1.063 - 1.321) | 0.002 |

**Table S3 Table of the log2-AISI multivariate model parameters**

| **Variable** | ***β*** | ***S.D*** | ***Wald χ²*** | **OR(95%CI)** | **P-value** |
| --- | --- | --- | --- | --- | --- |
| **Intercept** | -15.984 | 3.630 | 19.395 | - | <0.001 |
| log2-AISI | 1.151 | 0.299 | 14.819 | 3.162 (1.760 - 5.683) | <0.001 |
| Smoking status(Yes) | 0.569 | 0.565 | 1.013 | 1.767 (0.583 - 5.350) | 0.314 |
| Alcohol user(Yes) | 0.483 | 0.571 | 0.716 | 1.621 (0.529 - 4.962) | 0.398 |
| T-CHO(mmol/L) | 0.368 | 0.330 | 1.245 | 1.444 (0.757 - 2.755) | 0.265 |
| LDL(mmol/L) | 0.789 | 0.447 | 3.117 | 2.201 (0.917 - 5.283) | 0.077 |
| TP(g/L) | -0.052 | 0.027 | 3.713 | 0.950 (0.901 - 1.001) | 0.054 |
| CysC(mg/L) | 2.066 | 0.667 | 9.593 | 7.890 (2.135 - 29.156) | 0.002 |
| TPSA(ng/ml) | 0.171 | 0.062 | 7.474 | 1.186 (1.050 - 1.341) | 0.006 |
| FPSA(ng/ml) | 0.071 | 0.168 | 0.178 | 1.073 (0.773 - 1.491) | 0.673 |

**Table S4 Table of the optimized log2-AISI multivariate model parameters**

| **Variable** | ***β*** | ***S.D*** | ***Wald χ²*** | **OR(95%CI)** | **P-value** |
| --- | --- | --- | --- | --- | --- |
| Intercept | -15.103 | 3.384 | 19.916 |  | <0.001 |
| log2-AISI | 1.186 | 0.296 | 16.069 | 3.274(1.833 - 5.847) | <0.001 |
| LDL(mmol/L) | 1.101 | 0.311 | 12.559 | 3.007(1.636 - 5.529) | 0.000 |
| TP(g/L) | -0.052 | 0.026 | 4.047 | 0.949(0.903 - 0.999) | 0.043 |
| CysC(mg/L) | 2.090 | 0.626 | 11.129 | 8.081(2.368 - 27.582) | 0.001 |
| TPSA(ng/ml) | 0.169 | 0.056 | 9.294 | 1.184(1.062 - 1.321) | 0.002 |

**Table S5 Multicollinearity detection in the optimized log2-SII model**

| **Variable** | **TOL** | **VIF** |
| --- | --- | --- |
| log2-SII | 0.917 | 1.091 |
| Alcohol user(Yes) | 0.959 | 1.042 |
| LDL(mmol/L) | 0.900 | 1.111 |
| TP(g/L) | 0.897 | 1.115 |
| CysC(mg/L) | 0.889 | 1.125 |
| TPSA(ng/ml) | 0.899 | 1.112 |

TOL, Tolerance; VIF, Variance inflation factor

A VIF value more than 10 or a TOL less than 0.1 indicate multicollinearity.

**Table S6 Multicollinearity detection in the optimized log2-AISI model**

| **Variable** | **TOL** | **VIF** |
| --- | --- | --- |
| log2-AISI | 0.884 | 1.131 |
| LDL(mmol/L) | 0.890 | 1.124 |
| TP(g/L) | 0.907 | 1.103 |
| CysC(mg/L) | 0.896 | 1.116 |
| TPSA(ng/ml) | 0.903 | 1.107 |

TOL, Tolerance; VIF, Variance inflation factor

A VIF value more than 10 or a TOL less than 0.1 indicate multicollinearity.
