# Supplementary material for: Blood inflammatory markers combined with tumor markers for differentiating benign prostatic hyperplasia from prostate cancer
Source: Front Med (Lausanne). 2026 Feb 4;13:1730818. doi: 10.3389/fmed.2026.1730818 (PMC12913570; doi:10.3389/fmed.2026.1730818)
Supplement: Supplementary file 2 [file Supplementary_file_2.docx]

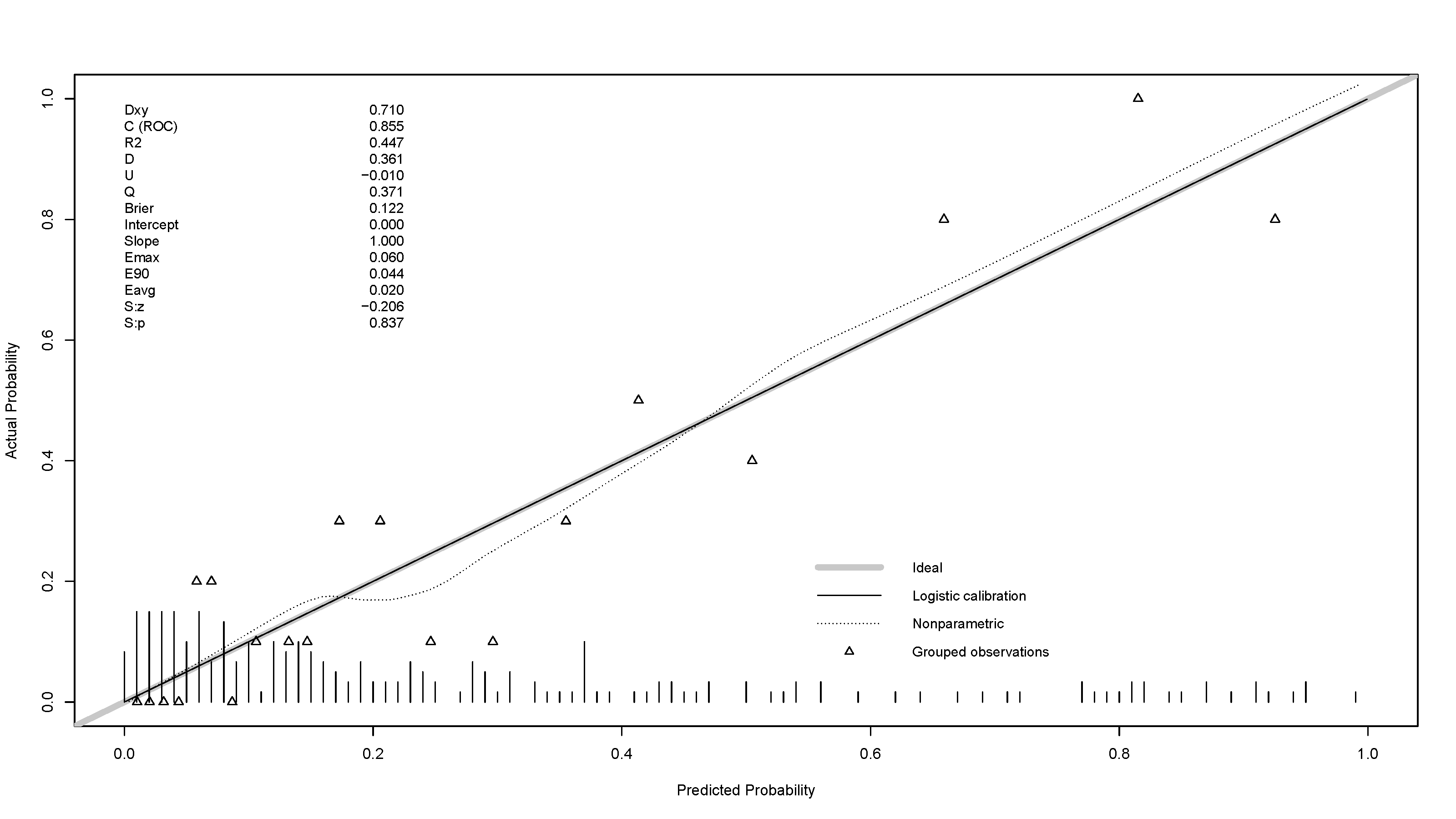


Figure S1. Calibration slope, intercept and Brier score of nomogram models of log2-SII.


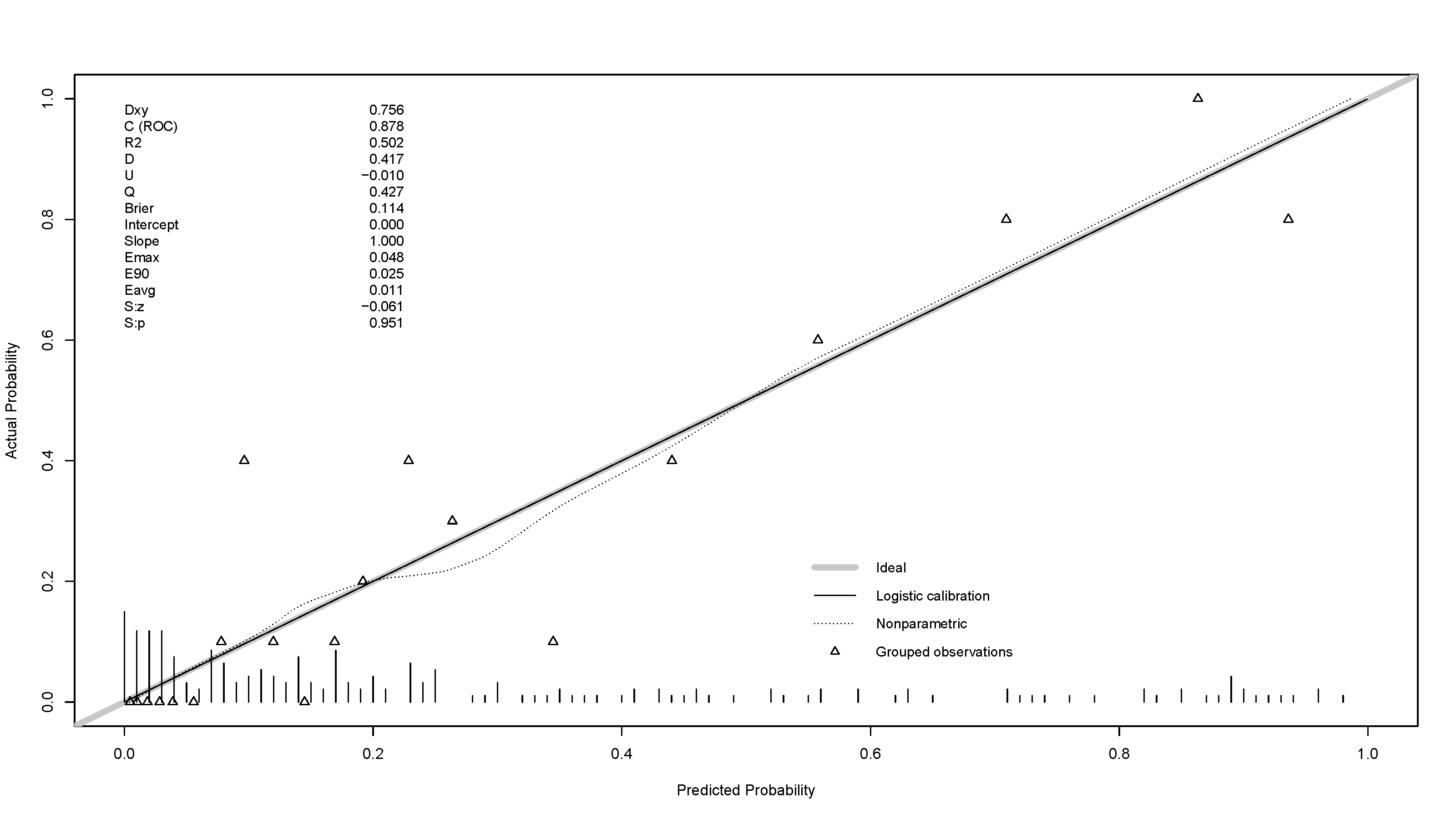


Figure S2. Calibration slope, intercept and Brier score of nomogram models of log2-AISI.


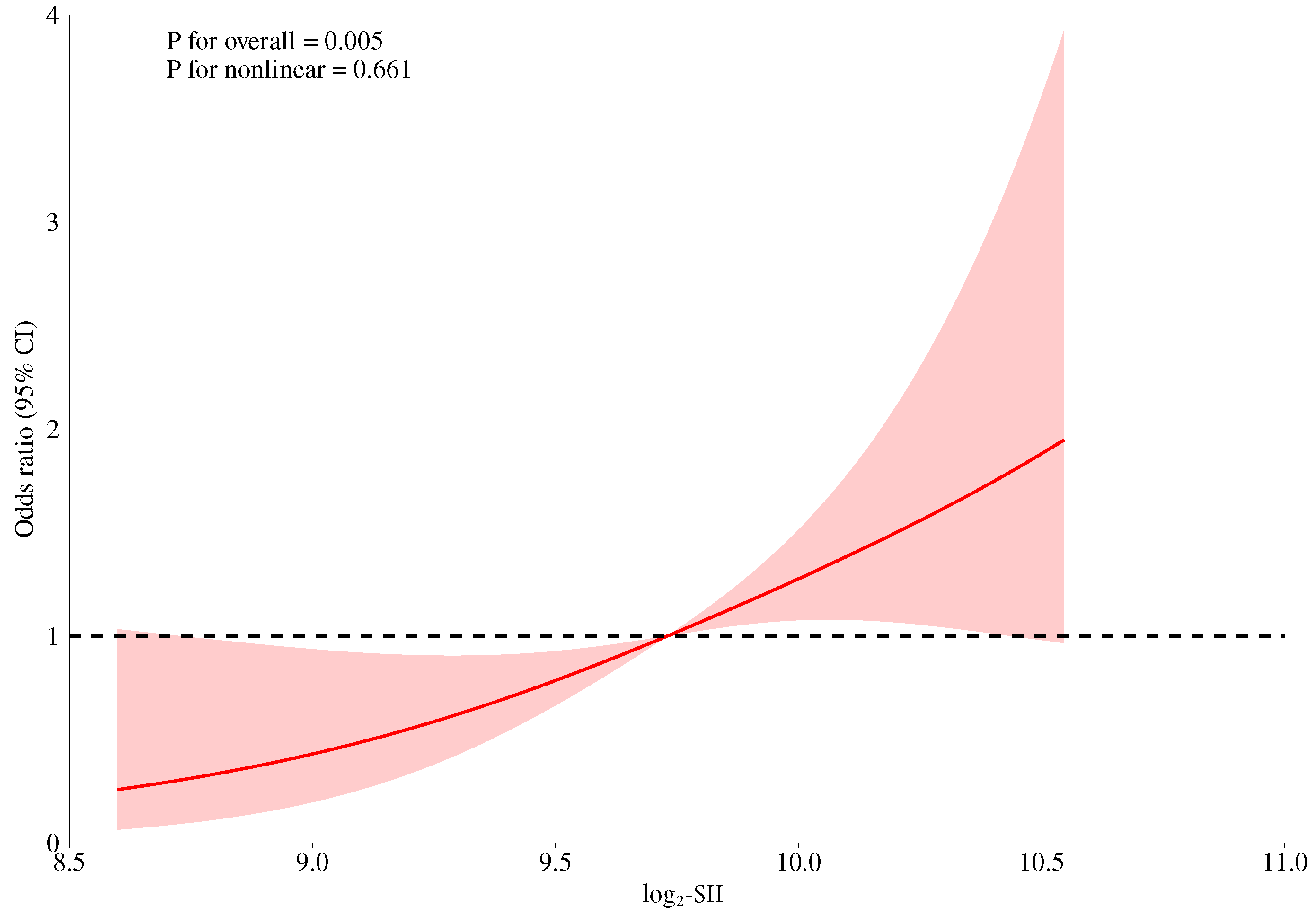


Figure S3A. RCS curve of the association between log2-SII and PCa


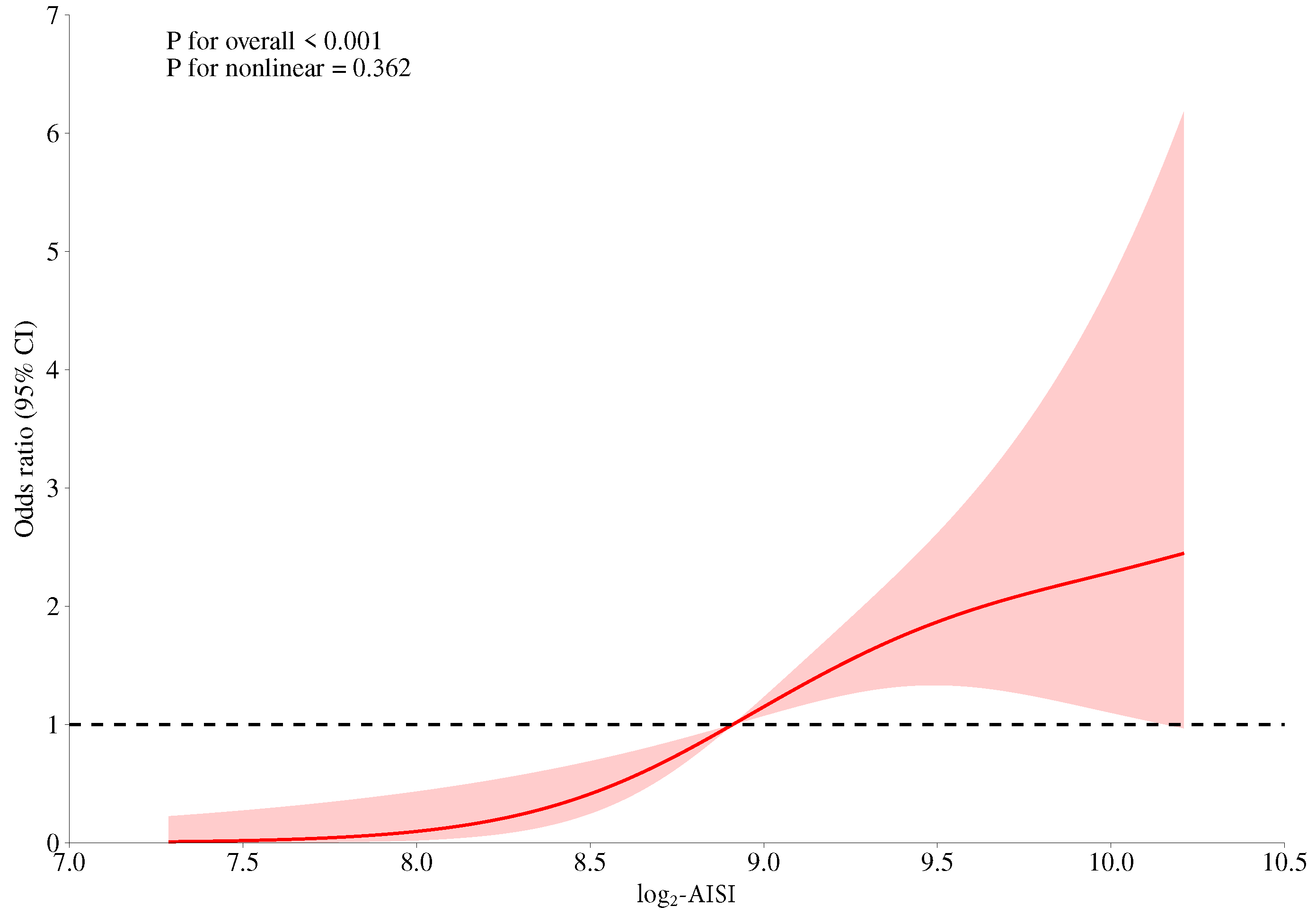


Figure S3B. RCS curve of the association between log2-AISI and PCa


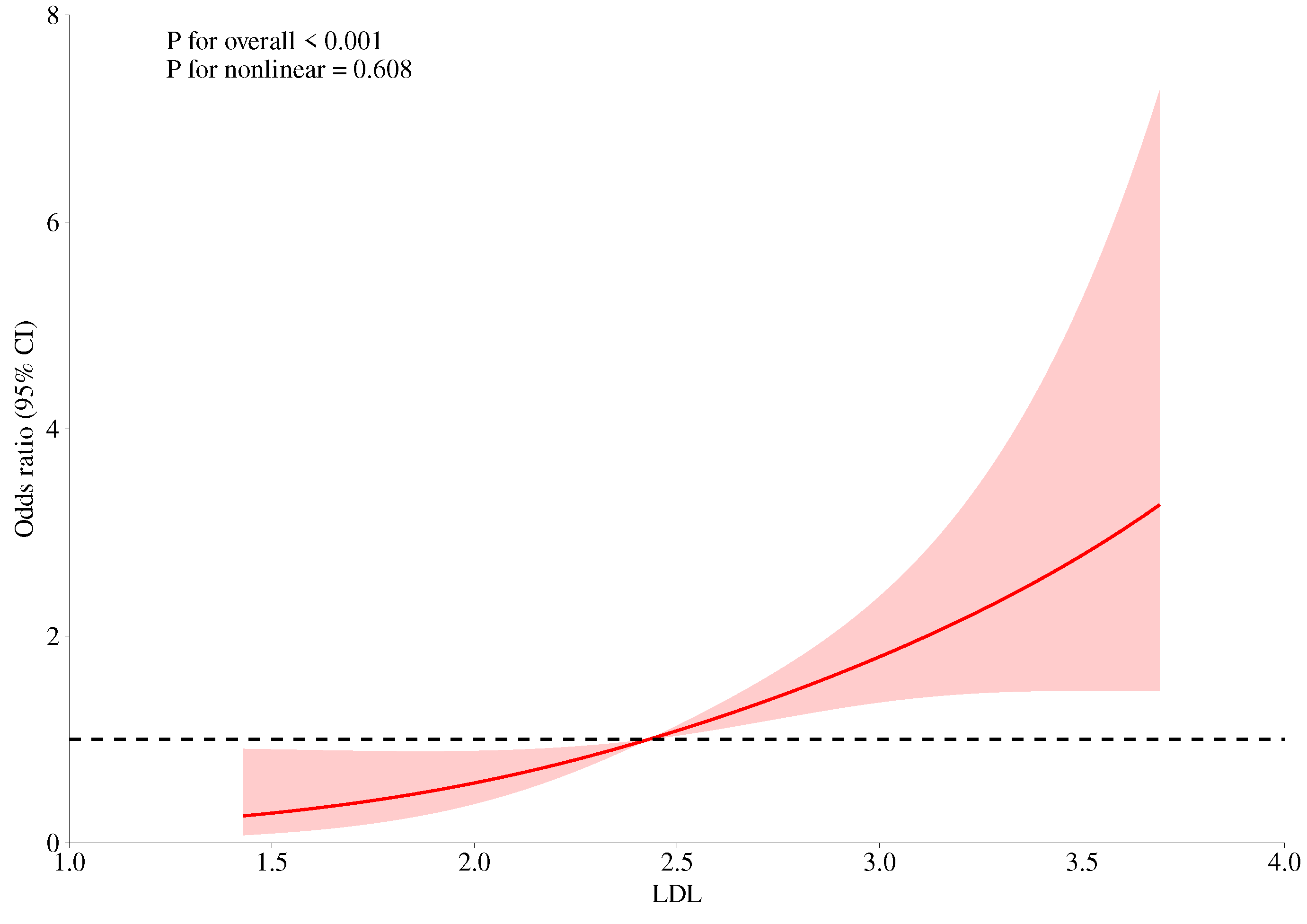


Figure S3C. RCS curve of the association between LDL and PCa


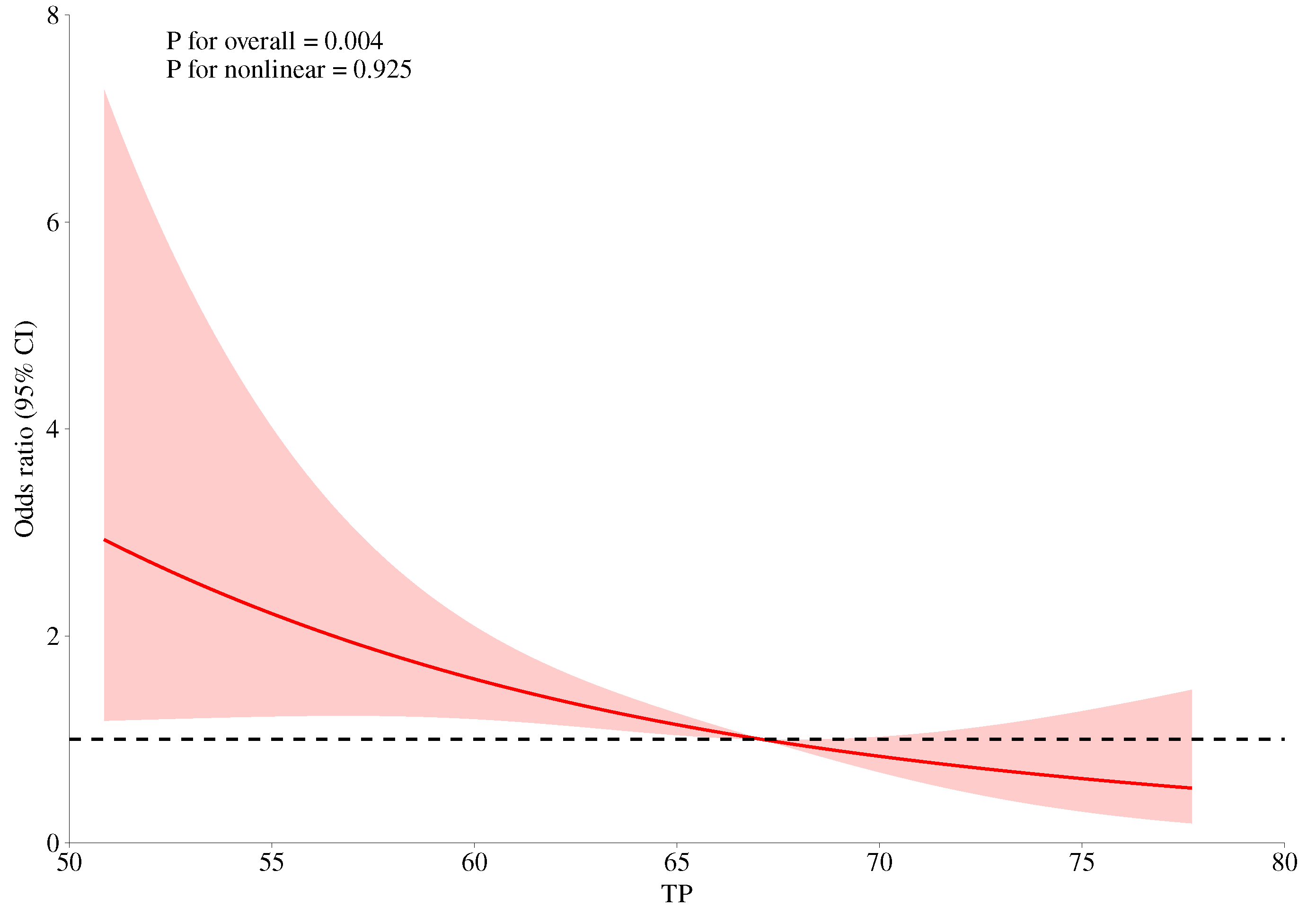


Figure S3D. RCS curve of the association between TP and PCa


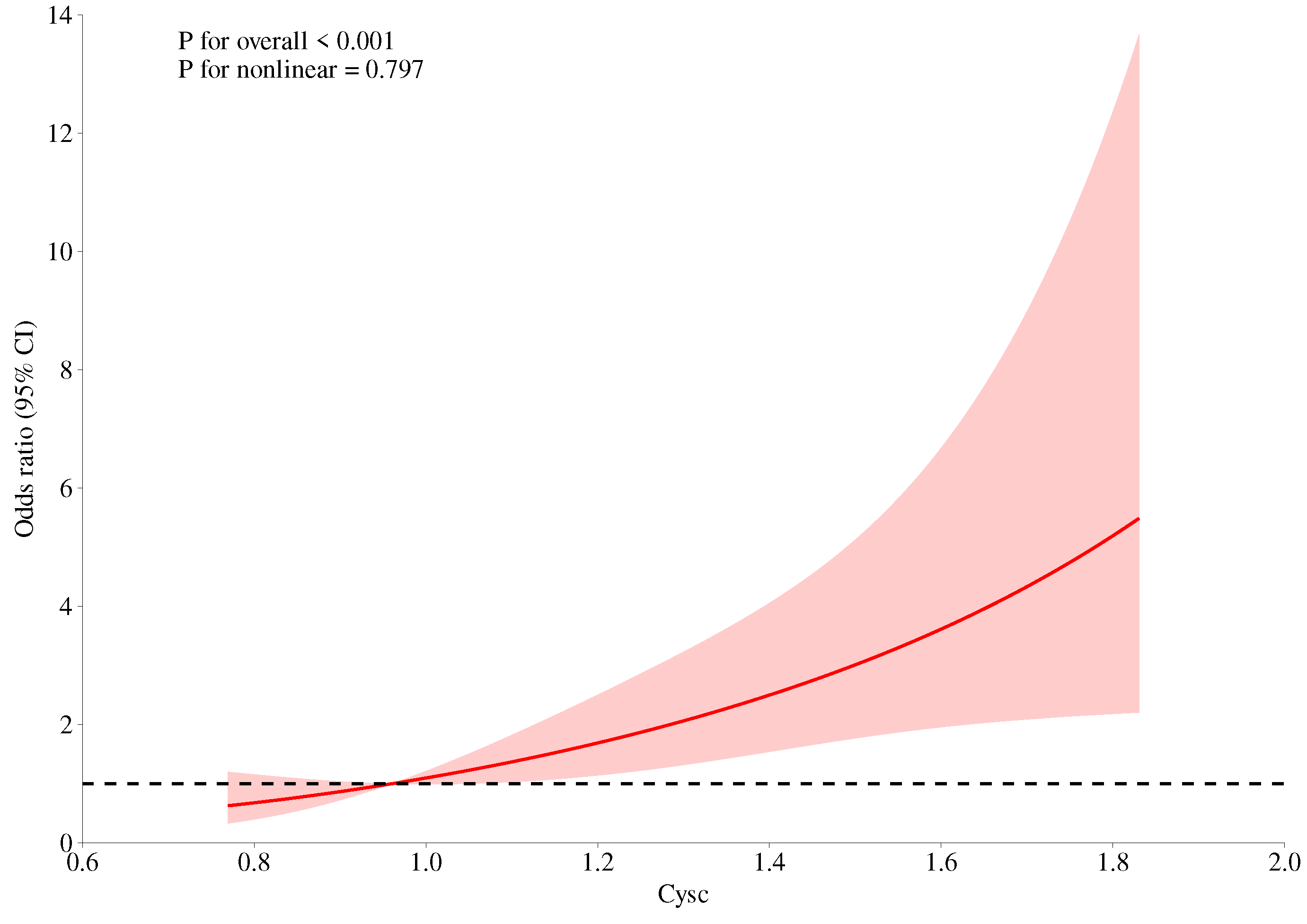


Figure S3E. RCS curve of the association between CysC and PCa


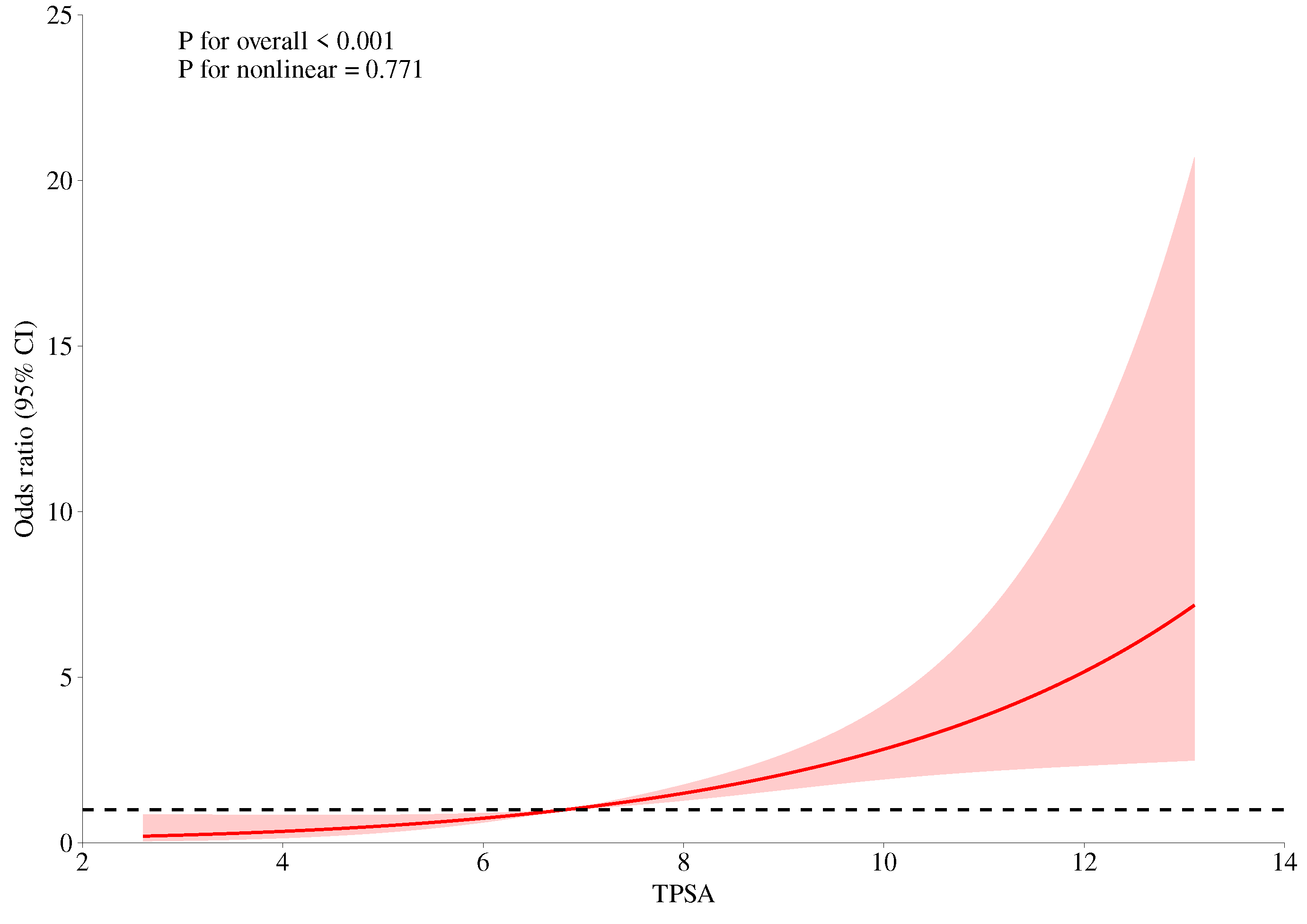


Figure S3F. RCS curve of the association between TPSA and PCa
